# Analysis of EEG signal by flicker-noise spectroscopy: identification of right-/left-hand movement imagination

**DOI:** 10.1007/s11517-016-1491-z

**Published:** 2016-04-08

**Authors:** A. Broniec

**Affiliations:** Faculty of Electrical Engineering, Automatics, Computer Science and Biomedical Engineering, AGH University of Science and Technology, al. A. Mickiewicza 30, Kraków, Poland

**Keywords:** Flicker-noise spectroscopy (FNS), Movement imagination (MI), Electroencephalography (EEG), Brain–computer interface (BCI)

## Abstract

Flicker-noise spectroscopy (FNS) is a general phenomenological approach to analyzing dynamics of complex nonlinear systems by extracting information contained in chaotic signals. The main idea of FNS is to describe an information hidden in correlation links, which are present in the chaotic component of the signal, by a set of parameters. In the paper, FNS is used for the analysis of electroencephalography signal related to the hand movement imagination. The signal has been parametrized in accordance with the FNS method, and significant changes in the FNS parameters have been observed, at the time when the subject imagines the movement. For the right-hand movement imagination, abrupt changes (visible as a peak) of the parameters, calculated for the data recorded from the left hemisphere, appear at the time corresponding to the initial moment of the imagination. In contrary, for the left-hand movement imagination, the meaningful changes in the parameters are observed for the data recorded from the right hemisphere. As the motor cortex is activated mainly contralaterally to the hand, the analysis of the FNS parameters allows to distinguish between the imagination of the right- and left-hand movement. This opens its potential application in the brain–computer interface.

## Introduction

Neuropsychological studies on processes occurring in the brain during the motor imagery (MI) show that similar parts of the brain are involved in the movement imagination as well as its real performance [11–13, 27]. The main difference between the movement execution and its imagination is that in the latter case, the movement performance is blocked at some level of the corticospinal information transfer [8–10, 24, 25]. This phenomenon is observed in the sport psychology, where many examinations show that mental exercises have a positive effect on the later movement execution [14, 19, 46]. Similarity between the movement imagination and the real movement was also confirmed by the research, in which healthy patients and patients with motor disabilities were subjected to the neuropsychological observation [16, 33]. This fact causes that the movement imagination plays an important role as a control signal in the brain–computer interfaces [62] which are dedicated to patients who partly or entirely lost the voluntary muscle contraction such as in the ’locked-in’ state [3, 28, 36, 45, 60]. A key factor in the successful design of the BCI systems is the method used to process and extract the meaningful information from the brain signal. The stage of signal processing in BCIs is comprised of the following components: preprocessing, feature extraction, feature selection and feature classification. The present paper deals with the second of them and shows that FNS can be an alternative method for the feature extraction in the analysis of the EEG signal related to the imagination of hand movement.

There are many methods used in the step of the feature extraction [1, 5, 6, 32, 37], depending on the type of the BCI systems, but interfaces which are based on the sensorimotor activity use mainly the power spectral density methods [35, 63], time–frequency representation [2, 17, 22, 64] or parametric modeling [7, 40]. All of them are based on the detection of characteristic rythms ($\mu$ or $\beta$) which form together the so-called resonant component of the signal. However, as we show in the paper, the information about the brain activity during the movement imagination is also contained in the chaotic component of the signal, and this information can be extracted from there by the use of the FNS method. The FNS is a time-series analysis method that introduces parameters characterizing the components of stochastic signals in different frequency ranges [59]. The method has found numerous applications; among others it is worth mentioning about the use of FNS to the parameterization of images produced by the atomic force microscopy (AFM) [51], analysis of geological signals measured in seismic areas [15, 49], determination of electric breakdowns precursors in thin porous silicon films [39], analysis of electric potential fluctuations in electromembrane systems [58] or monitoring cutting processes and development of stability maps for materials [29]. The FNS method was also successfully applied to some problems in a medical data processing. An example that is often quoted as evidence of this fact is the application of FNS in the analysis of the effect of different types of medical treatment on the dynamics of index finger tremor in Parkinsonian patients [65]. Moreover, Timashev et al. [52] used FNS for the identification of the photosensitive epilepsy. Their results suggest that FNS is a promising method of early diagnosis, not only for the photosensitive epilepsy but also for other neurodegenerative diseases such as Parkinson’s, Alzheimer’s, Huntington’s, amyotrophic lateral sclerosis and schizophrenia. These suggestions were confirmed in Ref. [55], where it was found that the FNS parameterization of EEG signal may be used for the diagnosis of schizophrenia at the early stages of its development.

Motivated by the successful applications of the FNS in the wide range of medical diagnoses, in the present paper we use this method to find episodes (extract the features) related to the imagination of the right- and left-hand movement. The aim of this paper is to show that FNS can be an alternative method for the features extraction in the BCI based on the sensorimotor activity. For this purpose, we performed an experiment consisting of 30 repetitions of right- or left-hand movement imagery trials, during which the EEG signal was recorded. Then, the signal (its chaotic component) was parametrized in accordance with the FNS method. We found the significant changes in the FNS parameters (visible as a peaks) at the time when the subject imagines the movement. The analysis carried out for the electrodes located on both hemispheres of the brain allows to observe that for the right-hand movement imagination an abrupt changes in the parameters appear for the data recorded from the left hemisphere, while for the left-hand movement imagination, the meaningful changes in the parameters are observed for the data recorded from the right hemisphere. Since the motor cortex is activated mainly contralaterally to the hand, the analysis of the FNS parameters allows to distinguish between the imagination of the right- and left-hand movement. This opens the potential application of the FNS method for the features extraction in the brain–computer interface (BCI).

## Methods

### Subjects and data acquisition

**Fig. 1:**
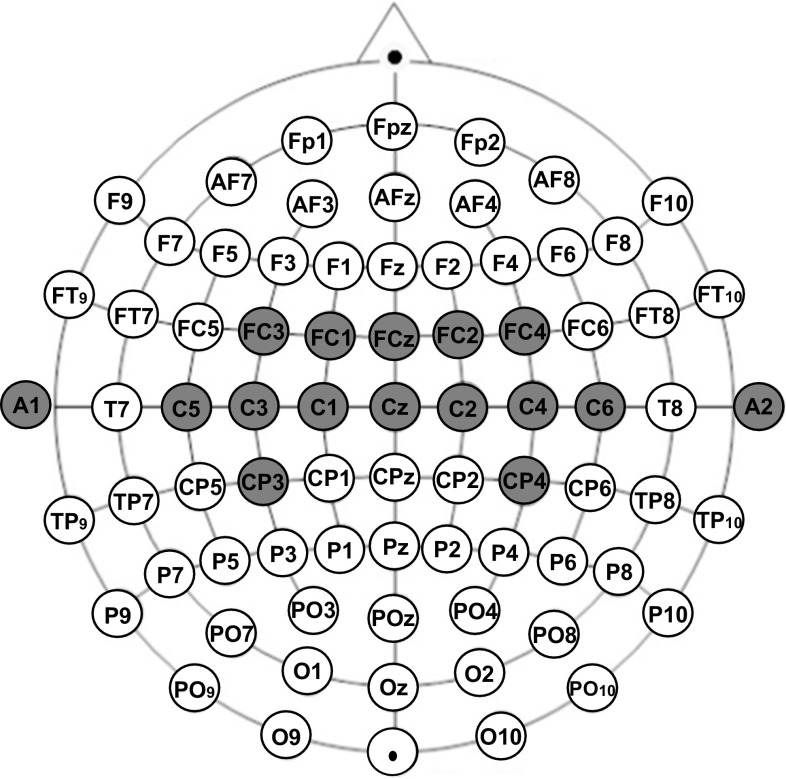
Extended international 10–20 system of the electrodes placement. The electrodes used for the data acquisition are marked by the *gray circles*. The ear’s electrodes $A_1$ and $A_2$ are the referencing ones

Nine volunteers (five females and four male) between the ages of 24 and 35 participated in this study. Eight of them are right-handed and one patient is bimanual. Only two of participants were experienced with MI tasks, and the others were naive subjects. All subjects gave informed consent. Moreover, one of the patients suffers from the spinal muscular atrophy (SMA). Each subject was seated in a comfortable armchair located about 1.5 m in front of a computer screen. Subjects were requested to relax the muscle and suppress eye blinking to avoid electromyographic (EMG) and electrooculographic (EOG) activity artifacts. When recording the EEG signal from the motor cortex, the strongest physiological artifacts stem from muscle movements, particularly from the neck contraction, face muscles contraction or swallowing. The visual inspection allows to effectively reject this kind of artifacts as they differ considerably, in the amplitude and the frequency, from EEG signal. The trials with evident artifacts were excluded, and only artifact-free EEG segments were used for the further analysis. Unipolar EEG channels were recorded from 14 gold disk electrodes placed over the left and right hemisphere over the cortical hand area according to the international extended 10–20 system (FC3, FC1, FCz, FC2, FC4, C5, C3, C1, Cz, C2, C4, C6, CP3, Cp4). The configuration of electrodes used for the data acquisition is shown in Fig. 1. Disk electrodes with electrode cream enable to keep the resistance between electrodes in the range $0.1-3.5\,\hbox {k}\Omega$. All 14 channels were referenced to the right or left ear’s lobe signals and ground from the forehead. Signals from all the channels were amplified with the biomedical signal amplifier g.USBamp (USB Biosignal Amplifier g.tec Guger Technologies). EEG signal was band-pass filtered (eighth-order Butterworth filter) between 0.5 and 100 Hz and recorded with a sample frequency of 1200 Hz.

**Fig. 2:**
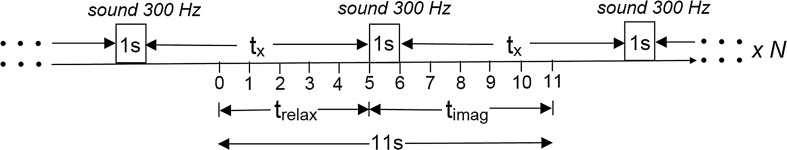
Schematic diagram of the experimental paradigm. The time interval taken for the analysis lasts 11 s and consists of two periods. First period, from 0 to 5 s, is the relaxation time $(t_{\mathrm{relax}})$ preceding the execution of the task. The second, from 5 to 11 s, which is indicated by the 1-s sound signal, is the time for the performance of the hand movement imagination and return to the relaxation state $(t_{\mathrm{imag}})$. $t_x$ is the time between the consecutive stimulus initiating the performance of the task. Its value varies randomly between 10 and 15 s

### Experimental paradigm

Since the MI is not a routine natural behavior in a daily life, its performance usually causes some difficulties [34]. The schematic diagram of the experimental paradigm is shown in Fig. 2. The experiment consists of 30 repetitions of right- or left-hand movement imagery trials. The duration time of the single trial takes 11 s and consists of two periods. First period, from 0 to 5 s, is the relaxation time $(t_{\mathrm{relax}})$ used as the referential time needed for calculating time–frequency maps. The second, from 5 to 11 s, which is indicated by the one-second sound signal, is the time for the performance of the hand movement imagination and return to the relaxation state $(t_{\mathrm{imag}})$. The duration time between the consecutive stimuli varies randomly but is not shorter than 8 s. This condition guarantees that the referential time is not disturbed by the expectation of the stimulus. In the experiment, the duration time $t_x$ between each stimulus is varied between 10 and 15 s. Each subject participated in at least two sessions with no feedback presented during the recordings. Each session consisted of 4–6 runs with 30 trials—half of them was for the right and half for the left-hand imagination. The number of runs depended on the tiredness which was subjectively assessed by the subject.

### Flicker-noise spectroscopy

The FNS method is based on the assumption that the information about the system is contained in “resonant” and “chaotic” components of the signal under study. According to the FNS method, the analyzed signal is separated into low-frequency component which corresponds to the specific “resonances” and two “chaotic” components having the source in signal’s irregularities such as spikes, jumps (discontinuities in the signal) and discontinuities in their derivatives of different orders [50]. Therefore, the considered signal *V*(*t*) (the EEG signal) can be written in the form

1 $$\begin{aligned} V(t)=V_{\mathrm{r}}(t)+V_{\mathrm{cS}}(t)+V_\mathrm{cR}(t), \end{aligned}$$

where $V_{\mathrm{r}}(t)$ is the resonant component, $V_{\mathrm{cS}}(t)$ is the chaotic component formed by spikes and $V_\mathrm{cR}(t)$ is the chaotic component formed by jumps.

The extraction of the information contained in the signal is performed by the power spectrum and the structural functions. Both of these functions are expressed in terms of the autocorrelation function which can be expressed in the form

2 $$\begin{aligned} \psi (\tau )=\frac{1}{T-\tau }\int _{0}^{T-\tau }V(t)V(t+\tau ) {\hbox {d}}t, \end{aligned}$$

where $\tau$ is the time lag $0\le \tau \le T/2$. The power spectrum (the cosine transform) of the autocorrelation function is defined as

3 $$\begin{aligned} S(f)=\int _{-T/2}^{T/2}\psi (\tau )\cos (2\pi f\tau )d\tau , \end{aligned}$$

The difference moment (structural function) of the second order $\phi ^{(2)}(\tau )$, (we assume $\left<V(t)\right>=0$), is given by

4 $$\begin{aligned} \phi ^{(2)}(\tau )=\frac{1}{T-\tau }\int _{0}^{T-\tau } {\left[ V(t)-V(t+\tau )\right] }^2 {\hbox {d}}t, \end{aligned}$$

which for stationary processes is simplified to the form [59]

5 $$\begin{aligned} \phi ^{(2)}(\tau )=2\left[ \psi (0)-\psi (\tau ) \right] \!. \end{aligned}$$

Since the stationarity condition is not fulfilled in real experiments, the whole duration of the experiment is divided into short time intervals in which it is assumed that the process is stationary.

### FNS parameterization

The FNS method allows to determine several parameters which describe the dynamics/characteristic of the system. All these parameters can be derived from the chaotic components of the functions *S*(*f*) and $\phi ^{(2)}(\tau )$ using the appropriate interpolation formulas presented underneath. The chaotic component of the difference moments $\phi ^{(2)}_c(\tau )$ can be approximated by [65]

6 $$\begin{aligned} \phi ^{(2)}_c(\tau )\approx 2\sigma ^2{\left[ 1-\varGamma ^{-1} \left( H_1,\frac{\tau }{T_1}\right) \right] }^2\!, \end{aligned}$$

where $\varGamma (s)=\varGamma (s,0)$, $\varGamma (s,x)=\int _{x}^{\infty } \exp (-t)t^{s-1}{\hbox {d}}t$ are the complete and incomplete gamma functions ($x\ge 0$ and $s>0$), $\sigma$ is the standard deviation of the measured variable, $H_1$ is the Hurst constant and $T_1$ is the correlation time. The approximation function for the chaotic power spectrum component $S_c (f)$ can be separated into two independent parts related to spikes $S_{\mathrm{cS}}(f)$ and jumps $S_\mathrm{cR}(f)$

7 $$\begin{aligned} S_{\mathrm{cS}}(f)=\frac{S_{\mathrm{cS}}(0)}{1+{\left( 2\pi fT_0 \right) }^{n_0}}, \end{aligned}$$

8 $$\begin{aligned} S_\mathrm{cR}(f)=\frac{S_\mathrm{cR}(0)}{1+{\left( 2\pi fT_1 \right) }^{2H_1+1}}, \end{aligned}$$

where $S_{\mathrm{cS}}(0)$, $n_0$, $T_0$ are the parameters and $S_\mathrm{cR}(0)$ is expressed as

9 $$\begin{aligned} S_\mathrm{cR}(0)=4\sigma ^2 T_1 H_1 \left\{ 1- \frac{1}{2H_1 \varGamma ^2(H_1)} \int _0^{\infty }\varGamma ^2(H_1,\xi )\hbox {d}\xi \right\} \!. \end{aligned}$$

Parameters introduced above have the following physical interpretation [59]: $\sigma$ is the standard deviation of the signal, time $T_1$ is the characteristic time interval after which the measured signal *V*(*t*) stops correlating, while the rate of the correlation loss is determined by the Hurst constant $H_1$. Parameters $S_{\mathrm{cS}}(0)$ and $S_\mathrm{cR}(0)$ characterize the boundary value of $S_{\mathrm{cS}}(f)$ and $S_\mathrm{cR}(f)$ in the low frequencies band, whereas $n_0$ describes the degree of the correlation loss in the frequency domain, when the frequency approaches to the value $1/T_0$. More details concerning the FNS method can be found in Refs. [53, 54, 56].

**Fig. 3:**
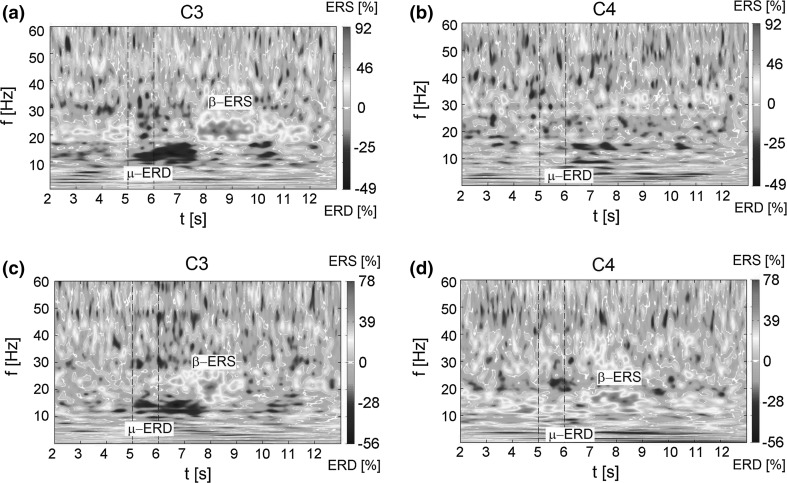
Maps of ERD/ERS in the time–frequency plane related to the right-hand movement imagination for electrode C3 (**a**) and electrode C4 (**b**) and the left-hand movement imagination for electrode C3 (**c**) and electrode C4 (**d**). On the *right side* of the graph, the scale of ERD/ERS changes expressed in percent. The *vertical dashed lines* mark the 1-s sound signal, after which the hand movement imagination and return to the relaxation state take place ($t_{\mathrm{imag}}$ in Fig. 2). The estimation of the time–frequency distribution of the energy density is scalogram. The reference period is 1–2 s

### ERD/ERS in the time–frequency plane

The classical way of describing both the execution and the imagination of the movement is the event-related desynchronization (ERD) and the event-related synchronization (ERS) [41–44]. ERD/ERS inform about the power decrease/increase in the brain activity, averaged over trials with respect to the power in a reference time interval. The standard method of calculating the ERD/ERS value [42] requires determining the frequency band at which the signal will be filtered and processing. However, the chosen band not always corresponds to the most significant changes in the power in the signal which can vary considerably between subjects. To obtain complete information in entire frequency range, the ERD/ERS in the time–frequency plane has to be evaluated in accordance with the formula [4]:

10 $$\begin{aligned} \hbox {ERD}/\hbox {ERS}(t,f)=\frac{\langle E(t,f) \rangle _{\mathrm{tr}}-B(f)}{B(f)}, \end{aligned}$$

where $\langle E(t,f) \rangle _{\mathrm{tr}}$ is the energy density at (*t*, *f*) averaged across trials and *B*(*f*) is the mean energy of baseline at frequency *f* averaged across trials. The method of estimating energy density of the signal was scalogram, which is based on the continuous wavelet transform (CWT) [18, 66]. CWT is defined as

11 $$\begin{aligned} W_{\mathrm{s}}(t,c)=\int _{-\infty }^{\infty } V(t') \frac{1}{\sqrt{c}} \varPsi ^{*}\left( \frac{t'-t}{c}\right) {\hbox {d}}t', \end{aligned}$$

where *c* is scale and *t* is translation. The Morlet’s wavelet was used

12 $$\begin{aligned} \varPsi (t)=\pi ^{-\frac{1}{4}} e^{- \frac{1}{2}t^2} e^{\mathrm{ikt}}, \end{aligned}$$

where *k* is an arbitrary choice constant—wavenumber.

The estimating energy density in the time-scale plane is given by

13 $$\begin{aligned} E(t,c)=|W_{\mathrm{s}}(t,c)|^2. \end{aligned}$$

This estimate is translated to the time–frequency plane leading to the expression

14 $$\begin{aligned} E(t,f)&=|W_{\mathrm{s}}(t,f)|^2 \nonumber \\&=\sqrt{\frac{2\sqrt{\pi }f}{k}}\left| \int _{-\infty } ^{\infty } V(t') e^{-\frac{1}{2} \left( \frac{2\pi f}{k} (t'-t) \right) ^2} e^{i2\pi f (t'-t)} {\hbox {d}}t' \right| ^2. \end{aligned}$$

In the paper, the time–frequency maps were calculated by the use of the open-source software [66] for $k=20$ which was chosen based on inspection of the raw ERD/ERS maps obtained for a number of different values of *k*.

## Results

In this section, first, we present the maps of ERD/ERS related to the hand movement imagination in the time–frequency plane. The ERD/ERS maps calculated by the continuous wavelet transform, presented in Sect. 2.5, are treated as the reference point for the FNS analysis. Next, the results of the FNS parameterization are presented, and finally, the changes in the FNS parameters as a function of time (during imagination of hand movement) are shown. Since the analysis of the data obtained for all subjects has yielded the consistent results with the exception of the individual characteristics, all figures in the paper present results for one of the subjects (S3). The EEG signal recorded during the experiment has been preprocessed for the ERD/ERS and FNS analysis in several steps. First, all trials with evident artifacts have been excluded. Second, the signal has been temporally filtered. Then, the signal has been spatially filtered using the small Laplacian filter, and finally, we have averaged the signal over all trials.

**Fig. 4:**
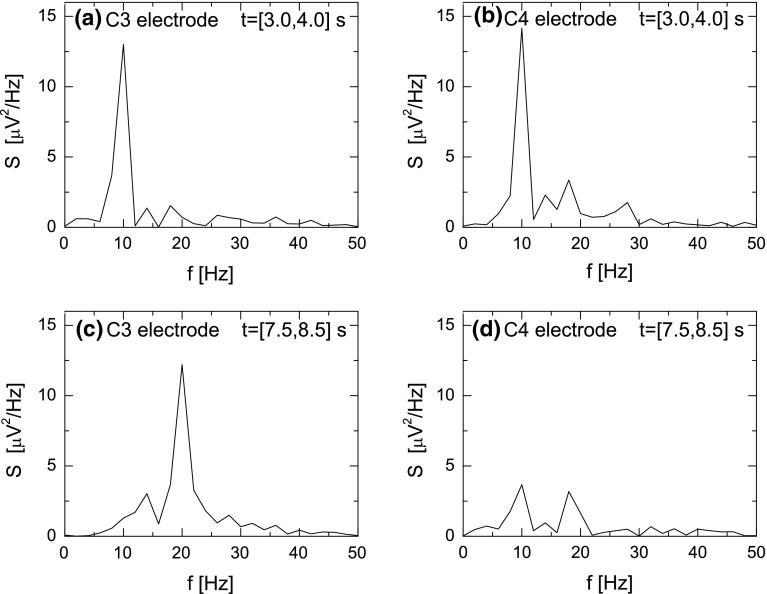
Linear-scale power spectrum *S*(*f*) of the EEG signal (the cosine transform of the autocorrelation function) calculated for two time intervals chosen during the experiment with the right-hand movement imagination. Graphs **a** and **b** for the time interval 3–4 s for electrodes C3 and C4, respectively, graphs **c** and **d** for the interval 7.5–8.5 s for electrodes C3 and C4

### Time–frequency maps of ERD/ERS related to the hand movement imagination

In case of experiment with hand movement, ERD appears in both the mu and beta bands before the movement (imagination), while ERS appears usually in the beta band as the post-movement beta synchronization ($\beta$-rebound). Since the human somatotopic organization indicates that human limbs are controlled by the contralateral brain hemispheres [21, 47, 48], we expect that the most important changes in the brain activity during the right-hand movement imagination occur mainly at electrode C3 which lies over the left hemisphere of the motor cortex whereas during the left-hand movement imagination at electrode laying over the right brain hemisphere, i.e., C4.

The ERD/ERS maps of the signal recorded during the imagination of the right-hand movement are presented in Fig. 3a, b at electrodes C3 and C4, respectively. Analogously, the ERD/ERS maps of the signal recorded at electrodes C3 and C4 during the imagination of the left-hand movement are presented in Fig. 3c, d. The vertical dashed lines mark the one-second sound signal, after which the hand movement imagination and return to the relaxation state take place ($t_{\mathrm{imag}}$). In Fig. 3a, b, one can observe that the $\mu$-ERD, before and during the imagination, appears bilaterally but mainly at the contralateral electrode C3. Simultaneously, in the range 20–30 Hz the contralateral synchronization ($\beta$-rebound) appears after the end of the task from 8 to 10 s. This phenomenon results from the synchronization of the neuron’s beta activity immediately after the termination of the task performance at the contralateral side of the brain. The ERD/ERS maps related to the imagination of the left-hand movement (see Fig. 3c, d) show that the $\beta$-rebound appears at electrode C4 (18 Hz) as well as the electrode C3 (about 20 Hz). Desynchronization in the $\mu$ range at the contralateral electrode is difficult to characterize and is only slightly outlined in the vicinity of 10 Hz. On the other hand, at electrode C3 desynchronization in this range is distinctly evident.

These results indicate that the changes in the synchronization of rhythms occur contralaterally only for the imagination of the movement with dominant hand, i.e., right for the considered subject. For the imagination of the movement with non-dominant hand, i.e., left, changes at the ipsilateral side (C3 electrode) have similar character as at the contralateral side (electrode C4). Therefore, the imagination of the movement with left hand, non-dominant for this patient, causes relatively similar changes in EEG signal at both electrodes. Their lateralization is not evident.

### FNS parameters extraction

The FNS method assumes that the signal under study is stationary. Since the human brain is a complex system generating non-stationary signal, this condition is not fulfilled. The solution to the problem is to check the dynamic of the parameters in the consecutive short time windows $[t_k,t_k+T]$ (where $k=0,1,2,3,\ldots$ and $t_k=k\Delta T$), shifted within the time limit of the total duration of the experiment [57]. It is assumed that in each window $[t_k,t_k+T]$ the signal is stationary. This procedure is analogous to the sliding window method applied in the classical technique of the signal processing. In order to determine the parameters according to the FNS methodology, the power spectrum *S*(*f*) (the cosine transform of the autocorrelation function) of the EEG signal has been calculated. In Fig. 4, the power spectra calculated using Eq. (3) are shown, for two different time intervals. Figure 4a, b presents the power spectra of the signal recorded in the time interval from 3 to 4 s at C3 and C4 electrodes, respectively. Since the stimulus initiates the movement imagination at 5th second, the chosen time interval is related to the preparation to the task execution. At both electrodes, the decreasing character of the EEG signal spectrum as a function of the frequency is visible with the distinct $\mu$ peak in 10 Hz. The peak corresponds to the dominance of the $\mu$ wave before the task execution. The $\mu$ rhythm is then reduced with intention to move and can be observed as ERD in the time–frequency maps (see Fig. 3). In Fig. 4c, d, the power spectrum of the signal recorded from 7.5 to 8.5 s at C3 and C4 electrodes is presented. In this time interval, the strong dominance of the $\beta$ rhythm at the C3 electrode in 20 Hz can be observed. This corresponds to the contralateral synchronization ($\beta$-rebound) visible in the time–frequency maps after the end of the task (see Fig. 3).

**Fig. 5:**
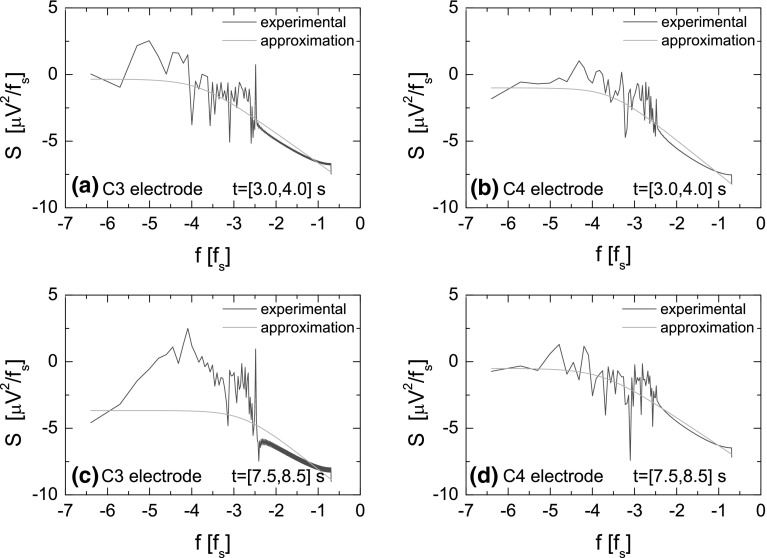
Log–log-scale power spectra *S*(*f*) of the EEG signal determined for the same data as in Fig. 4. In *blue*—the power spectra for the experimental data, in *green color*—the approximation of the chaotic component calculated using the function $S_{\mathrm{cS}}(f)$ (see Eq. 7) (color figure online)

**Fig. 6:**
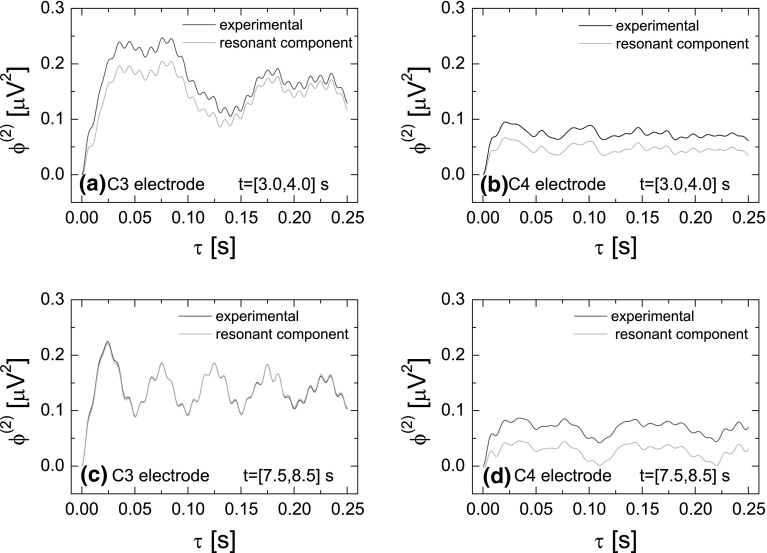
The difference moments $\phi ^{(2)}(\tau )$. **a**–**d** are presented for the same data as in Fig. 4. The function $\phi ^{(2)}(\tau )$ calculated for experimental data is presented by the *blue line* while the *green line* displays the resonant component $\phi _{\mathrm{r}}^{(2)}(\tau )$ (color figure online)

**Fig. 7:**
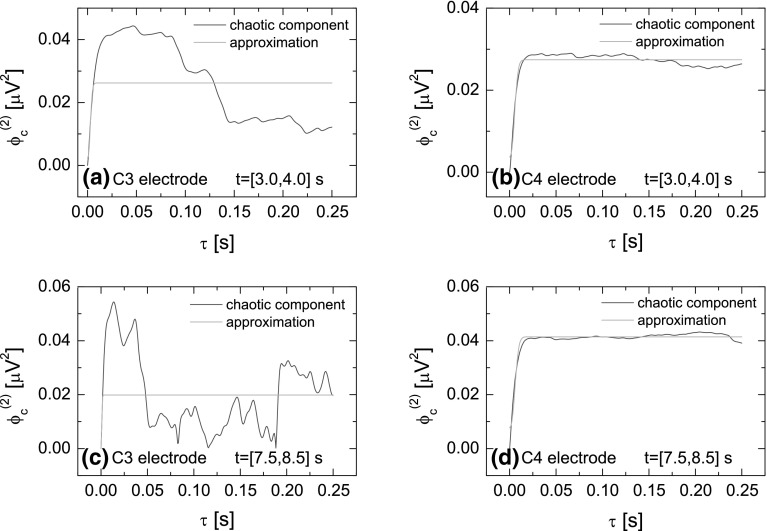
Chaotic component of the difference moments $\phi _c^2(\tau )$ (*blue line*) and its approximation (*green line*) determined using the formula (6). Results are presented for the same data as in Fig. 4 (color figure online)

The peaks on the power spectra (Fig. 4) are formed by the resonant component of the signal $V_{\mathrm{r}}(t)$ which contains the main information about the brain activity including the characteristic rythms. These correspond to the ERD/ERS maps usually calculated to illustrate the brain activity during the movement imagination (Fig. 3). Nevertheless, as we will show below, the information about the brain activity is also contained in the chaotic component of the EEG signal which can be extracted therefrom by the use of the FNS method. This method assumes that the information hidden in the chaotic component of the signal can be expressed by a set of parameters which are determined by the approximation of the chaotic power spectrum and difference moments using the formulas (6)–(9) presented in Sect. 2.4. Below, we briefly describe the methodology of the FNS parameters extraction from the EEG signal recording during the experiment.

First, the power spectra *S*(*f*) of the EEG signal are presented in the log–log scale as in Fig. 5. This procedure suppresses the peaks originating from the resonant component and makes the numerical approximation of the chaotic one much easier. Figure 5a–d is presented for the same data as shown in Fig. 4. The parameters $S_{\mathrm{cS}}(0)$, $T_ 0$ and $n_0$ can be extracted from the signal by the approximation of the power spectrum *S*(*f*) in the log–log scale using the formula given by Eq. (7). In Fig. 5, the blue curves correspond to the power spectrum of the experimental data while their approximation is displayed by the green color. The approximation has been performed numerically by the least squares method.

The parameters $H_1$, $T_1$ and $\sigma$ are determined based on the difference moments $\phi _c^{(2)}(\tau )$ of the chaotic component of the signal (see Eq. 6). The function $\phi _c^{(2)}(\tau )$ is calculated by the subtraction of the difference moments $\phi ^{(2)}(\tau )$ for the experimental data from the difference moments $\phi _{\mathrm{r}}^{(2)}(\tau )$ for the resonant component of the signal. The latter is determined from the resonance component of the autocorrelation function $\psi _{r}(\tau )$ calculated by applying the inverse Fourier transformation of the power spectrum $S_{\mathrm{r}}(f)=S(f)-S_{\mathrm{cS}}(f)$. In other words, the subtraction of the chaotic component approximation $S_{\mathrm{cS}}(f)$ (Eq. 7) from the experimental spectra *S*(*f*) gives the power spectra corresponding to the resonant component $S_{\mathrm{r}}(f)$ which inverse Fourier transformation gives the resonant autocorrelation function $\psi _{r}(\tau )$ needed to calculate $\phi _{\mathrm{r}}^{(2)}(\tau )$ (see Eq. 5). In Fig. 6, the function $\phi ^{(2)}(\tau )$ calculated for the experimental data is displayed by the blue lines, while the resonant component $\phi _{\mathrm{r}}^{(2)}(\tau )$ of the difference moment is marked by the green color. In Fig. 7, the function $\phi _c^{(2)}(\tau )$ (blue line) and its approximation (green line) by the formula (6) are presented. As previously, the approximation has been carried out numerically with the least squares method. This procedure allows to determine parameters $H_1$, $T_1$, $\sigma$ and indirectly, using the formula (9), the parameter $S_\mathrm{cR}(0)$.

**Fig. 8:**
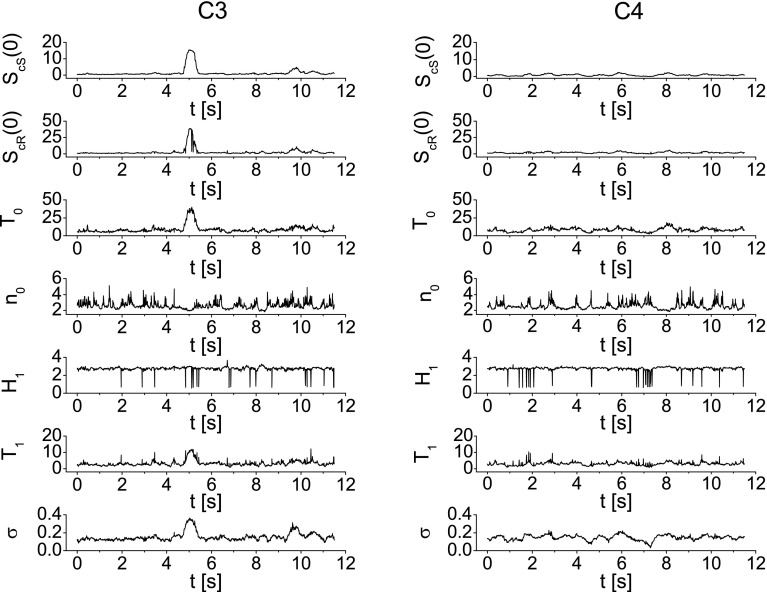
Values of the parameters $S_{\mathrm{cS}}(0)$, $S_\mathrm{cR}(0)$, $T_0$, $n_0$, $H_1$, $T_1$, $\sigma$ as a function of time for the imagination of the right-hand movement. Parameters have been calculated for the time window $T=0.5$ s ($N=600$) moved along the whole duration of the experiment. The *left column* shows the values of parameters for electrode C3 and *right* for electrode C4

**Fig. 9:**
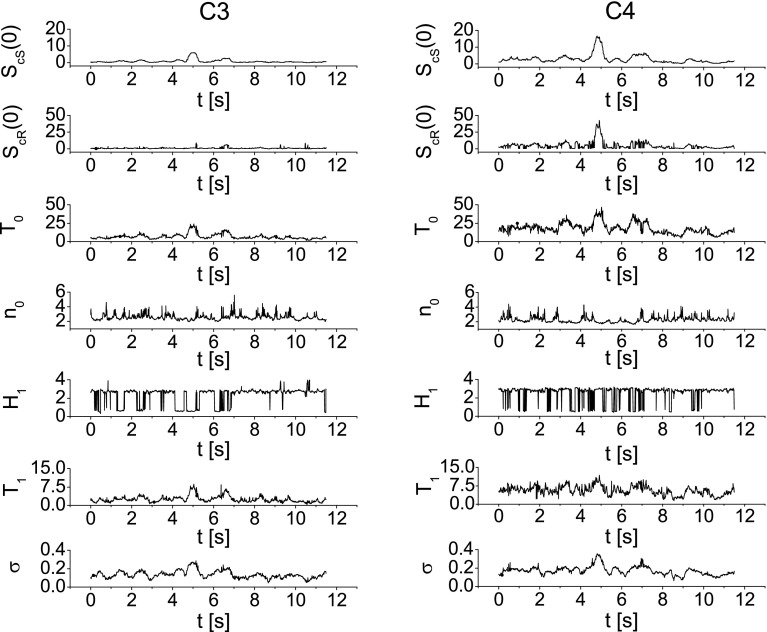
Values of the parameters $S_{\mathrm{cS}}(0)$, $S_\mathrm{cR}(0)$, $T_0$, $n_0$, $H_1$, $T_1$, $\sigma$ as a function of time for the imagination of the left-hand movement. Parameters have been calculated for the time window $T=0.5$ s ($N=600$) moved along the whole duration of the experiment. The *left column* shows values of parameters for electrode C3 and *right* for electrode C4

### Analysis of FNS parameters related to right-/left-hand movement imagination

**Fig. 10:**
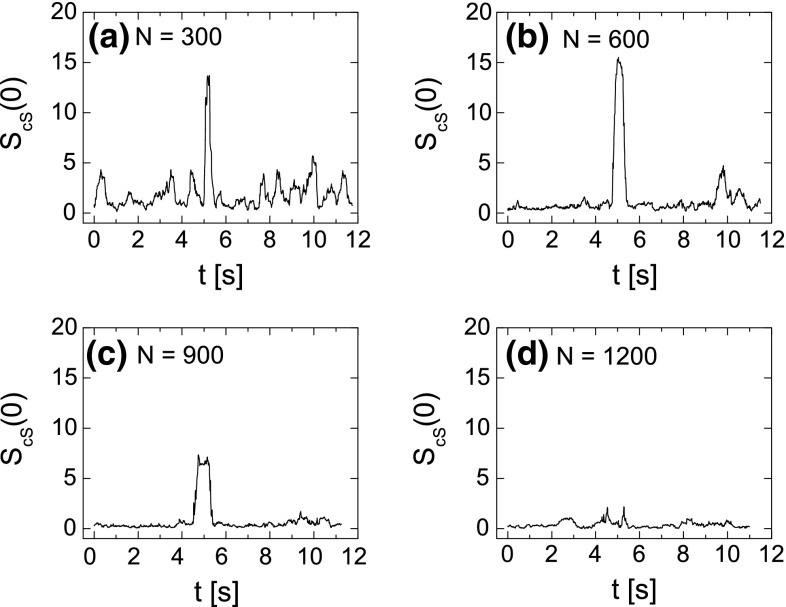
Values of the parameter $S_{\mathrm{cS}}(0)$ as a function of time for the imagination of right-hand movement (electrode C3). Parameter has been calculated for four different time windows, i.e., **a** $T=0.25$ s ($N=300$), **b** $T=0.5$ s ($N=600$), **c** $T=0.75$ s ($N=900$) and **d** $T=1$ s ($N=1200$)

In order to determine the changes in the FNS parameters in time, their values have been considered in the constant time window $T=0.5$ s (which is equivalent to $N=600$ samples) moved along the whole duration of the experiment. For each window position, the parameters $S_{\mathrm{cS}}(0)$, $S_\mathrm{cR}(0)$, $T_0$, $n_0$, $H_1$, $T_1$, $\sigma$ have been calculated with the procedure described above. Figure 8 displays the values of the FNS parameters as a function of time during the imagination of the right-hand movement. Left column presents the parameters calculated for the data recorded at C3 electrode, whereas in the right column at electrode C4. We can observe that the values of the parameters vary in time. Figure 8 shows that for the data recorded at electrode C3 an abrupt changes (visible as a peak) in the parameters $S_{\mathrm{cS}}(0)$, $S_\mathrm{cR}(0)$, $T_0$, $\sigma$ appear at about 5th second, which corresponds to the initial moment of the movement imagination. In contrary, at electrode C4, no meaningful changes in the parameters are observed at this moment. Since the changes related to the right-hand movement imagination should predominantly occur at electrode C3, which lies over the left hemisphere above the area of the motor cortex, this finding agrees with our expectations. In Fig. 9, the changes in the FNS parameters as a function of time are shown during the imagination of the left-hand movement. Significant increase in the parameter values $S_{\mathrm{cS}}(0)$, $S_\mathrm{cR}(0)$, $T_0$, $T_1$, $\sigma$ at about 5th second is observed at C4 electrode which lies over the motor areas contralateral to the left hand. At the ipsilateral electrode C3, the values of parameters $S_{\mathrm{cS}}(0)$, $T_0$, $T_1$, $\sigma$ also increase at this moment, but this enhancement is lower comparing to the one observed at C4 electrode. This phenomenon is also observed in ERD/ERS maps (see Fig. 3). It seems to be characteristic that for the subjects changes related to the movement imagination of the right hand occur mainly at the contralateral electrode C3. At the same time, the left-hand movement imagination induces changes mainly at electrode C4. Table 1 shows the ratio of the $S_{\mathrm{cS}}(0)$ peak amplitude for the electrode C3 to $S_{\mathrm{cS}}(0)$ amplitude for the electrode C4 for all nine subjects. It can be seen that for the right-hand movement imagination the ratio is greater than 1, while for the left-hand imagination is less then 1. Based on the presented results, we can conclude that the investigation of the FNS parameters as a function of time reveals the moment of the task execution and allows to distinguish between imagination of right- and left-hand movement. One has to note that the length of the time window *T* can affect the results. As the main tool to extract the information contained in the signal is the autocorrelation function, it causes that the information is averaged over the whole given time window *T*. This means that the longer the time window is, the more information is lost as an effect of averaging. In order to show this effect, in Fig. 10 the parameter $S_{\mathrm{cS}}(0)$ as a function of time for imagination of right-hand movement (at electrode C3) is shown. Calculations have been done for four different time windows, i.e., (a) $T=0.25$ s ($N=300$), (b) $T=0.5$ s ($N=600$), (c) $T=0.75$ s ($N=900$) and (d) $T=1$ s ($N=1200$). We can see that the extension of the time window *T* above $N=1200$ points (which corresponds to $T=1$ s) causes a decay of the $S_{\mathrm{cS}}(0)$ peak and as a consequence loss of the information about the movement imagination.

**Table 1:** The ratio of the $S_{\mathrm{cS}}(0)$ peak amplitude for the electrode C3 to $S_{\mathrm{cS}}(0)$ amplitude for the electrode C4 for all nine subjects

Subject	$S_{\mathrm{cS}}^{C3} (0)/S_{\mathrm{cS}}^{C4} (0)$	Hand
S1	0.12	Left
	1.3	Right
S2	0.69	Left
	1.54	Right
S3	0.7	Left
	1.37	Right
S4	0.8	Left
	1.29	Right
S5	0.84	Left
	1.38	Right
S6	0.17	Left
	8.24	Right
S7	0.47	Left
	1.73	Right
S8	0.81	Left
	1.17	Right
S9	0.7	Left
	1.46	Right

## Discussion

In the present paper, we have shown that the FNS can be applied for the analysis of electroencephalography signal related to the hand movement imagination. Our approach proved to be successful because parameterization of the signal faultless indicates the moment of the movement imagination as well as the hemisphere activated by the task (see Figs. 8, 9). As we have shown, the motor cortex is activated mainly contralaterally to the hand, namely the left side of the brain is responsible for controlling the right side of the body, while the right hemisphere is responsible for control of the left side of the body. This can be quantitatively described by the ratio of the $S_{\mathrm{cS}}(0)$ peak amplitude for the electrode C3 to $S_{\mathrm{cS}}(0)$ amplitude for the electrode C4, as shown in Table 1. It can be seen that for the right (left)-hand movement imagination the ratio is greater (less) than 1. This allows of effective differentiation between right- and left-hand movement imagination.

Since the motor imagery is not a routine natural behavior in a daily life, its performance usually causes some difficulties. Moreover, subject’s individual characteristics have considerable influence on the data. This individual characteristics are evident at the ERD/ERS maps which are treated as the reference point in our study. The character of these features mainly depends on: subject’s training with MI tasks, the dominance of the hemisphere and the sex [20, 31, 38]. In the preliminary analysis of the ERD/ERS maps, only three out of nine participants showed changes in the motor rhythms, as expected. Only two of participants were experienced with MI tasks, and the others were naive subjects. Six out of seven subjects who participated in such an experiment for the first time presented no significant changes during MI. It can be expected that feedback and subject training with MI tasks would help these subjects to increase their performance [31]. The dominance of the hemisphere can be explained by the environmental influence. Since the right-handedness is most common, right-handed people are less forced by everyday situations to use the left hand, and their right area of the motor cortex is less trained. In contrast, left-handed persons, by the same environmental influence, are encouraged to use the right, non-dominant hand [20, 61]. The great majority of left-handed persons demonstrate similar fitness for the performance of motor tasks with both hands. Results presented in Ref. [20] confirm the general feature that the population of left-handed persons are characterized by a smaller morphological and functional asymmetry of the cerebral cortex than right-handers. Wittelson [61] showed that left-handers have a bigger corpus callosum than right-handers which potentially facilitate bilateral activation of cerebral hemispheres. Moreover, the literature reports that the differences in the brain activity of right- and left-handers appear during experiments consisting of pressing the button [26, 30]. Additionally, it is worth mentioning that according to Okada’s research [38] the majority of women use both hemispheres while performing the motor task, whereas the majority of men make a movement using the hemisphere which is dominant. Thus, the sex is an additional factor which determines the character of ERD/ERS changes.

An exciting finding of this study is that the results of the analysis of the data by the FNS method show significant peak at the moment of MI for each of nine subjects. Moreover, the amplitude of peaks at the right and left hemisphere clearly indicates which hand participated in the task. Despite the differences in peak height for each subject, the ratio $S_{\mathrm{cS}}^{C3}(0)/S_{\mathrm{cS}}^{C4}(0)$ remains independent of subject’s individual characteristics as given in Table 1. The main conclusion from these findings is that the FNS parameterization is promising for the potential BCI application. In the step of the feature extraction, BCI interfaces, which are based on the sensorimotor activity, use mainly the power spectral density methods, time–frequency representation or parametric modeling. They base on the alpha and beta band (8–35 Hz) activity. As we shown, the FNS method can be ranked to the parametric modeling, but its novelty is that the information about the brain activity is extracted from the chaotic component of the signal. The FNS parameters can serve as features used for classification. Thus, FNS provides a new solution in features extraction or can support already existing solution in BCI.

Our research focused on the application of the FNS methodology to the analysis of the movement imagination task, and to the best of our knowledge, this is the first proposal of the FNS analysis of EEG signal related to the episodes which are used as the BCI paradigm. Although the results of the analysis are encouraging, practical experience indicates that further improvements are needed. Our future work will explore which FNS parameters produce the best result for classification and how many least trials are needed for good feature extraction. Other promising field of exploration could be BCIs based on the FNS parameters.

## Conclusions

Nowadays, the EEG-based BCI systems have reached an asymptotic trend in the accuracy of performance; however, it is still characterized by a significant error rate [23, 40]. A chance to fulfill a dream about the BCI that is easy to use and helpful for disable persons in everyday activity requires technological and methodological breakthroughs. To improve the accuracy and efficiency of the BCI systems, a new signal processing techniques and other innovative solutions need to be tested. These are the reasons why we have investigated the FNS method as an alternative for the analysis of EEG signal related to the motor imagery.

In this paper, the FNS has been used for the analysis of the EEG signal related to the movement imagination. Under experimental conditions, nine subjects performed several repetitions of either left- or right-hand motor imagery. The signal has been parameterized in accordance with FNS method. Significant increase in parameters values (visible as a peaks) related to the task execution has been observed. As shown in the paper, the parameterization of the signal with FNS method clearly indicates the time point of movement imagination as well as the hemisphere activated by the task which allows for effective differentiation between right- and left-hand movement imagination. Since this differentiation is crucial for the potential application in the BCI, one can conclude that the FNS method could be a potential method for the features extraction in the BCI systems based on the sensorimotor activity.
